# Selective Inhibitory Effect of Epigallocatechin-3-gallate on Migration of Vascular Smooth Muscle Cells

**DOI:** 10.3390/molecules15118488

**Published:** 2010-11-19

**Authors:** Dong-Wook Han, Mi Hee Lee, Byeong-Ju Kwon, Hye-Lee Kim, Suong-Hyu Hyon, Jong-Chul Park

**Affiliations:** 1 Department of Nanomedical Engineering, College of Nanoscience & Nanotechnology, Pusan National University, Busan 609-735, Korea; E-Mail: nanohan@pusan.ac.kr (D-W.H.); 2 Brain Korea 21 Project for Medical Science, Cellbiocontrol Laboratory, Department of Medical Engineering, Yonsei University College of Medicine, Seoul 120-752, Korea; E-Mails: LEEMH1541@yuhs.ac (M.H.L.); bjlove123@yuhs.ac (B.J.K.); haley@yuhs.ac (H.L.K.); 3 Department of Medical Simulation Engineering, Research Center for Nano Medical Engineering, Institute for Frontier Medical Sciences, Kyoto University, Kyoto 606-8507, Japan; E-Mail: biogen@frontier.kyoto-u.ac.jp (S-H.H.)

**Keywords:** eigallocatechin-3-gallate, vascular endothelial cells, vascular smooth muscle cells, proliferation, migration

## Abstract

In order to prevent restenosis after angioplasty or stenting, one of the most popular targets is suppression of the abnormal growth and excess migration of vascular smooth muscle cells (VSMCs) with drugs. However, the drugs also adversely affect vascular endothelial cells (VECs), leading to the induction of late thrombosis. We have investigated the effect of epigallocatechin-3-gallate (EGCG) on the proliferation and migration of VECs and VSMCs. Both cells showed dose-dependent decrease of viability in response to EGCG while they have different IC_50_ values of EGCG (VECs, 150 μM and VSMCs, 1050 μM). Incubating both cells with EGCG resulted in significant reduction in cell proliferation irrespective of cell type. The proliferation of VECs were greater affected than that of VSMCs at the same concentrations of EGCG. EGCG exerted differential migration-inhibitory activity in VECs *vs.* VSMCs. The migration of VECs was not attenuated by 200 μM EGCG, but that of VSMCs was significantly inhibited at the same concentration of EGCG. It is suggested that that EGCG can be effectively used as an efficient drug for vascular diseases or stents due to its selective activity, completely suppressing the proliferation and migration of VSMCs, but not adversely affecting VECs migration in blood vessels.

## 1. Introduction

Intimal hyperplasia leading to restenosis is the major process that limits the success of cardiovascular intervention. Neointimal hyperplasia in response to arterial injury is a complex process, classically believed to be the consequence of vascular smooth muscle cell (VSMC) proliferation and migration, and the synthesis of extracellular matrix [1]. The neointimal lesion induced by vascular injury evolves as a result of the synergistic interplay between the endothelial cell (EC) layer, platelets and VSMCs [2]. Direct injury to vascular cells is an important stimulus for the production of autocrine-paracrine growth factors, such as PDGF-AA, TGF-β1, basic fibroblast growth factor and insulin-like growth factor I, that promote the proliferation of VSMCs, whereas blood-borne factors (e.g., PDGF-BB and thrombin) appear to be more important in promoting the migration of VSMCs into the subintimal space [3,4,5]. There have been reported various therapeutic methods including chemotherapy, brachytherapy and gene therapy in order to suppress intimal hyperplasia [6,7,8]. However, some treatments were effective in *in vitro* or animal studies, whereas other studies have found little or no benefit in clinical applications [9].

Polyphenols with strong antioxidant properties, derived from diverse dietary foods or beverages such as tea, grape, turmeric, *etc*, have shown cancer chemopreventive and chemotherapeutic effects in many cell culture systems and animal tumor bioassays [10,11,12]. Among various nutraceutical ingredients, epigallocatechin-3-gallate (EGCG), the most abundant and most active catechin derivative in green tea, predominantly accounts for the biological effects of green tea [10]. Epidemiological and experimental studies have established a positive correlation between the consumption of green tea and protection against atherosclerosis and cardiovascular diseases [13]. The underlying mechanisms of action involve vascular and myocardial effects of tea constituents [14]. Mechanisms that have been suggested as being involved in the antiatherosclerotic effects of green tea consumption primarily entail antioxidative, anti-inflammatory, antiproliferative and antithrombotic properties, as well as beneficial effects on endothelial function [13,14,15]. Many *in vitro* and *in vivo* studies have shown that green tea polyphenolic compounds (GTPCs) has potent antioxidative, radical-scavenging metal ions-chelating, and redox-sensitive transcription factors-inhibiting properties, which may partially account for their antiatherogenic effects [15,16,17].

In the present study, we investigated the effect of EGCG on the cellular behaviors of vascular cells, ECs and SMCs although they were originated from different species, *i.e.* human and rat, respectively. Our data exhibit that the proliferation of both cells is dose-dependently decreased in response to increasing concentrations of EGCG, while the migration responses to EGCG are completely different between VECs and VSMCs. This differential effect of EGCG may be exploited to develop strategies for the prevention of neointima formation and subsequent restenosis by EGCG.

## 2. Results and Discussion

### 2.1. Immunocytochemical Characterization of VSMCs

**Figure 1:**
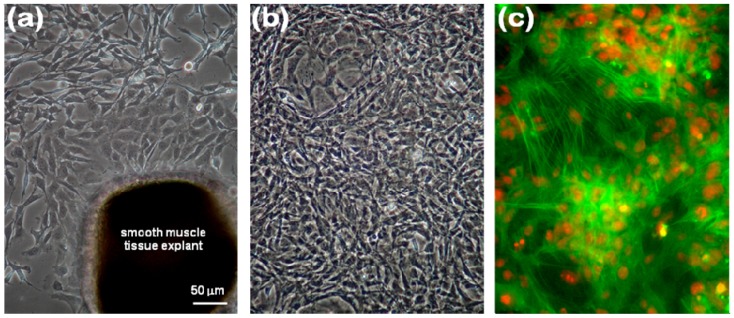
Optical microscopic photographs of primarily cultured VSMCs, **(a)** growing out of smooth muscle tissue explant of rat inferior vena cava (at 5 d after isolation) and **(b)** cultured for 4 d at passage 4. **(c)** Fluorescence microscopic photograph of VSMCs cultured for 2 d at passage 5, immunostained with FITC–SMA (green) and counterstained with PI (red) for nuclei. The micrographs (×200) shown in this figure are representative of six independent experiments, showing similar results.

Figure 1(a) shows VSMCs growing out of the tunica media of rat inferior vena cava. The cells were cultured for 5 d after isolation and became confluent after further 2 d of cultivation. While the confluent cells were sub-cultured, the tissue explants were removed by washing with PBS. Through several passages, the remaining cells were further sub-cultured [Figure 1(b)]. The initial shape of the isolated cells was fibroblast-like, but at over 90% confluent condition, the cell shape became more spindle-like, showing that they were in the process of differentiation to SMCs [18]. At passage 5, the primarily cultured cells were immunostained for α-smooth muscle actin (SMA), well-known marker for SMCs differentiated from myofibroblasts, together with nucleus counterstaining by propidium iodide (PI). In Figure 1(c), FITC-conjugated SMA was intensively observed in the cells, suggesting that the cells had been differentiated into SMCs [18].

### 2.2. Cytotoxicity Profiles of EGCG to VECs and VSMCs

**Figure 2:**
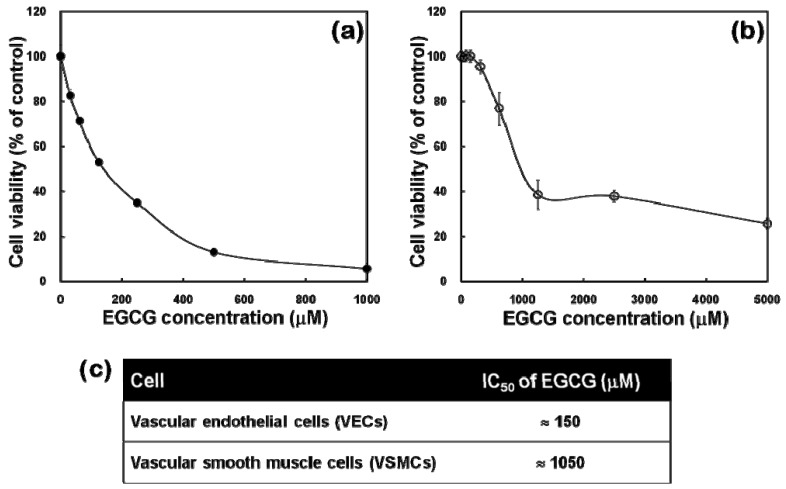
Cytotoxicity profiles of EGCG against cultured VECs **(a)** and VSMCs **(b)**. Cytotoxicity was measured by CCK-8 assay, as described in Experimental section. **(c)** IC_50_ values of EGCG to VECs and VSMCs were determined from their respective cytotoxicity profiles.

As shown in Figure 2(a), the relative viability of VECs treated with increasing concentrations (0 ~ 1,000 μM) of EGCG was decreased in a dose-dependent manner (from 31.3 μM EGCG). On the contrary, the cytotoxicity profile (0 ~ 5,000 μM EGCG) of VSMCs was quite different from that of VECs. They were still viable even at over 300 μM EGCG and exhibited an abrupt decrease in the cell viability from 625 μM (Figure 2B). It was also revealed and the IC_50_ values of EGCG to VECs and VSMCs were determined as approximately 150 μM and 1,050 μM, respectively [Figure 2(c)].

### 2.3. Effects of EGCG on Proliferation of VECs and VSMCs

**Figure 3:**
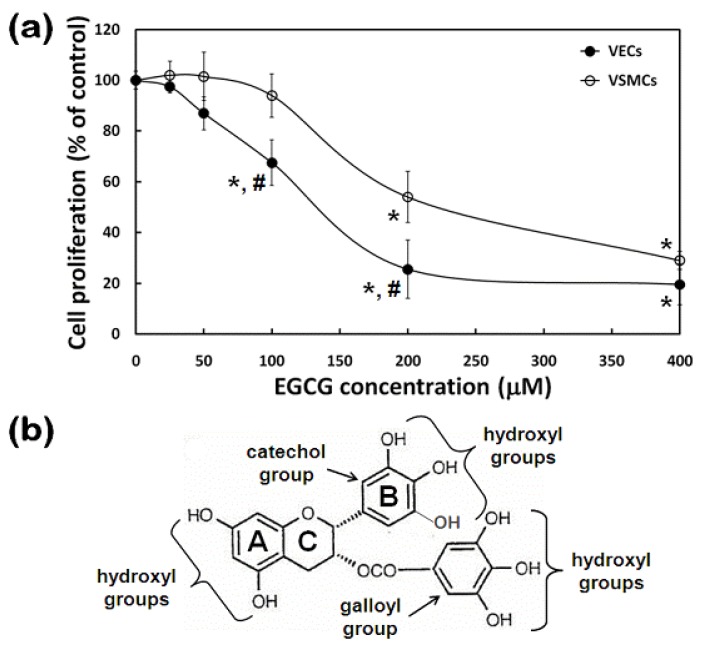
**(a)** Effects of EGCG on proliferation of VECs and VSMCs. **(b)** Chemical structures of EGCG, the main polyphenolic constituents of green tea.

To investigate whether EGCG renders the proliferation of FBS-stimulated both types of vascular cells attenuated or not, their proliferation patterns in response to EGCG were examined. Incubating the cells with EGCG resulted in significant (*p* < 0.05) reduction in cell growth irrespective of cell type [Figure 3(a)]. The proliferation of VECs were greater affected than that of VSMCs at the same concentrations of EGCG, suggesting that EGCG exerts potent antiproliferative activity attributed to its molecular structure with para hydroxyl and galloyl groups responsible for antioxidant effects (Figure 3B) [19]. Our previous study related to the antioxidative activity of GTPCs had already demonstrated that oxidative stress induced by reactive oxygen species was effectively prevented by GTPC treatment in human microvascular ECs [20]. A previous study regarding cancer therapy reported that EGCG had selective anti-angiogenic effects on tumor-associated ECs and endothelial progenitor cells [21].

VSMC proliferation and neointima formation are important events in the pathophysiological course of atherosclerosis and restenosis after balloon angioplasty. After endothelial cell activation, locally produced growth factors and cytokines mediate an inflammatory response within the vessel wall, which involves monocyte recruitment, stimulation of macrophage proliferation, migration of VSMCs from the medial layer of the vessel and finally deposition of collagen and other extracellular matrix proteins leading to the formation of a fibrous cap [22]. EGCG has been shown to have protective effects on the cardiovascular system, including anti-atherosclerotic and anti-hypercholesterolemic effects [23]. A number of studies have reported that EGCG inhibit VSMC proliferation and hypertrophy and block the stimulation by serum or growth factors [24,25]. In VSMCs, EGCG has been shown to inhibit the angiotensin II-induced phosphorylation of ERK 1/2, JNK 1/2, or p38 MAPK [26] and to reduce increases in cytosolic free Ca^2+^ concentration induced by angiotensin II [27].

### 2.4. Differential Effects of EGCG on Migration of VECs vs. VSMCs

**Figure 4:**
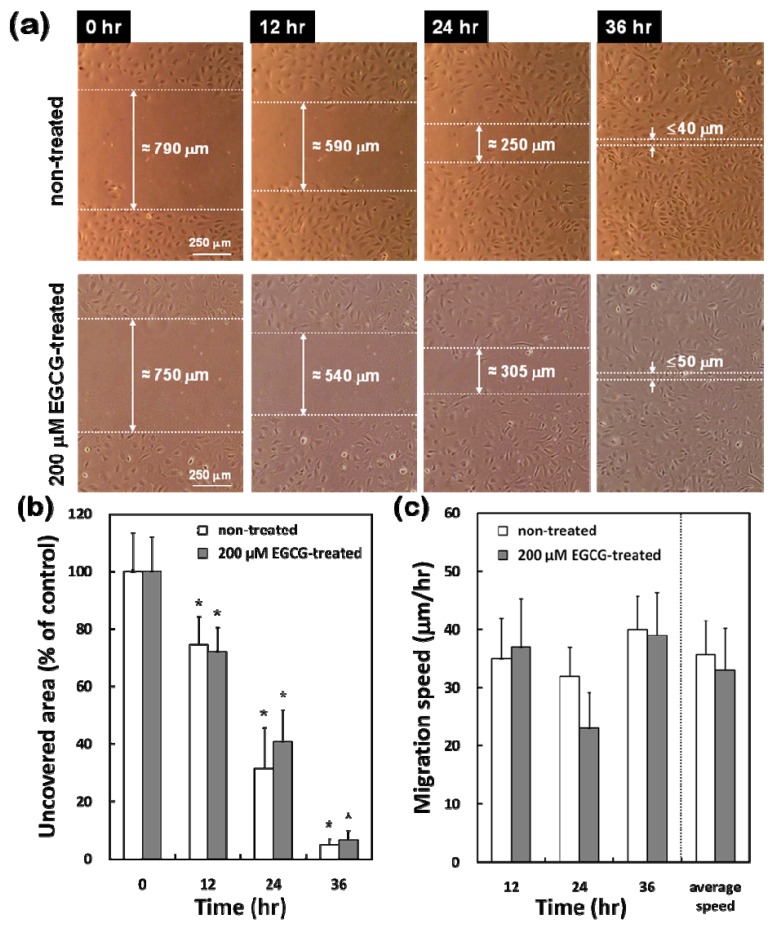
Effect of EGCG on migration of VECs. **(a)** Microscopic photographs (×40) of cells migrating into denuded areas (representative of six independent experiments, showing similar results). Uncovered area **(b)** and migration speed **(c)** were calculated by image processing software. The data are reported as means ± SD (n = 6) and analyzed by a Tukey HSD test. The values marked with an asterisk are significantly different from 0 hr (* *p* < 0.05).

*In vitro* migration assays revealed that although VECs were exposed to EGCG with sufficient concentration and duration (200 μM and up to 36 hr), the recovery of the scraped area was almost completed by the migrating cells covering the denuded space at 36 hr regardless of EGCG treatment [Figure 4(a)]. The initial denuded area of non-treated cells was 0.864 ± 0.115 mm^2^, and about 5 % of the area remained uncovered after 36 hr of incubation with 35.7 ± 5.9 μm/hr of the average migration speed [Figure 4(b) and Figure 4(c)]. In the case of EGCG-treated VECs, the denuded area closure showed appreciably similar pattern to that of non-treated cells except for slightly reduced migration speed at 24 hr. The denuded area was gradually reduced with the progress of the incubation period to about 7% of initial area showing 33.0 ± 7.3 μm/hr of the average migration speed.

**Figure 5:**
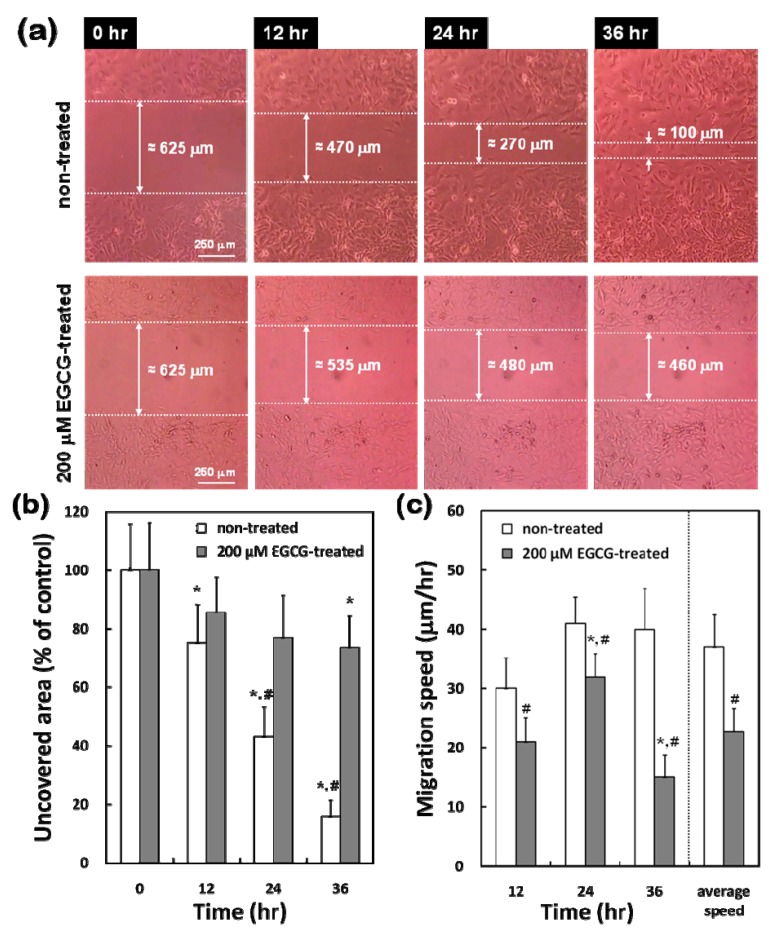
Effect of EGCG on migration of VSMCs. **(a)** Microscopic photographs (×40) of cells migrating into denuded areas (representative of six independent experiments, showing similar results). Uncovered area **(b)** and migration speed **(c)** were calculated by image processing software. The data are reported as means ± SD (n = 6) and analyzed by a Tukey HSD test. The values marked with an asterisk are significantly different from 0 hr or average speed (* *p* < 0.05). The values marked with a sharp are significantly different between non-treated and EGCG-treated at the same time (# *p* < 0.05).

On the contrary, the migration pattern of VSMCs in response to 200 μM EGCG was quite different from that of VECs. The denuded area in EGCG-treated cells showed appreciably delayed closure and still remained unpopulated through 12 to 36 hr, even though cells at the edge of the scrape showed forward movement [Figure 5(a)]. The first evidence of cell ingrowth in EGCG-treated VSMCs did not appear until around 12 hr and scrape recovery was not complete until sometime between 24 hr and 36 hr after EGCG treatment. Non-treated VSMCs normally migrated into the scraped space after 36 hr of denuding leaving uncovered area of about 16% with 37.0 ± 5.5 μm/hr of the average migration speed [Figures 5(b) and 4(c)].

Since the doubling time for these VSMCs with FBS stimulation was about 36 ~ 40 hr (our unpublished observation), the recovery in non-treated cells at 36 hr implied that the cells had extensively migrated from the edge of the scrape into the denuded space prior to the onset of cell proliferation. However EGCG-treated cells did not show any migration prior to the onset of cell proliferation and ingrowth was delayed even after the onset of cell proliferation due to EGCG. The EGCG-treated cells covered only 26% of the initial scraped area even after 36 hr. There was significant (*p* < 0.05) difference in the uncovered area and migration speed between the non-treated and EGCG-treated cells at 24 hr and 36 hr.

The proliferation of VECs showed a dose-dependent inhibition in response to EGCG like VSMCs, while VECs migration did not. These peculiar phenomena of VECs to EGCG could not be clearly elucidated here. However, it seems to be in part explained by the evidence that the type of matrix metalloproteinases (MMPs) directly involved in cell migration was completely different between VECs and VSMCs although cell cycle progress closely related to cell proliferation was negatively affected by EGCG in both cells [28]. MMP-2 and MMP-9 play key roles in migration and matrix invasion of VSMCs as gelatinase A and gelatinase B, respectively [29]. MMP-1 is closely involved in VECs migration as a collagenase [30]. In the case of MMP-13, it is mainly related to macrophage stimulation in vulnerable human atheromatous plaques by increasing collagenolysis as interstitial collagenases-1 and -3 [31].

## 3. Experimental

### 3.1. Cell Cultures

Animal care followed the criteria of the Animal Care Committee of Yonsei University College of Medicine for the care and use of laboratory animals in research. All experiments related to surgical procedures and treatments were performed in accordance with the guidelines of the Animal Experiment Committee of Yonsei University College of Medicine. VSMCs were isolated by limited enzymatic digestion from the tunica media of inferior vena cava of male young adult (9 ~ 10 wk old) Sprague-Dawley rats (280 ~ 300 g in weight, Samtako Inc., Gyeonggi-do, Korea) as previously reported [32,33]. The primarily cultured VSMCs were maintained in a complete RPMI 1640 medium containing 11.1 mM D-glucose (Sigma–Aldrich Co., St. Louis, MO, USA), supplemented with 10% fetal bovine serum (FBS, Sigma–Aldrich) and 1% antibiotic antimycotic solution (including 10,000 units penicillin, 10 mg streptomycin and 25 μg amphotericin B per ml, Sigma–Aldrich) at 37 °C and 5% CO_2_ in a humid environment. Studies were performed with the cells between third to fifth passage.

VECs were purchased from Lonza (human umbilical vein ECs, Walkersville, MD, USA) and cultured in endothelial cell basal medium-2 containing 11.1 mM D-glucose (Lonza) supplemented with 2% FBS (Lonza), EC growth supplements (Lonza; hEGF, hydrocortisone, hEGF, VEGF, hFGFB, R3-IGF-1 and ascorbic acid) and 1% antibiotic antimycotic solution (Sigma–Aldrich) at 37 °C in a humidified atmosphere of 5% CO_2_ in air as previously reported [34]. Studies were performed with the cells between passages 3 to 5.

### 3.2. Morphological and Immunocytochemical Analyses of Primarily Cultured VSMCs

On day 5 after seeding the minced smooth muscle tissue explant onto a culture flask and on day 4 after the forth passage, the morphologies of the primarily cultured cells were respectively observed using a phase-contrast microscope (TE 300, Nikon, Osaka, Japan). Prior to seeding VSMCs into well plates, the cells at the fifth passage were characterized by a standard immunostaining method where mouse monoclonal anti-α-SMA antibody conjugated with FITC isomer I (Sigma–Aldrich) and PI (Sigma–Aldrich) for nucleus counterstaining were combined with fluorescence microscopy (Biozero – 8000, Keyence, Osaka, Japan) as previously described [32,33].

### 3.3. Cytotoxicity of EGCG to VECs and VSMCs

The number of viable cells was quantified indirectly using a highly water soluble tetrazolium salt [WST-8, 2-(2-methoxy-4-nitrophenyl)-3-(4-nitrophenyl)-5-(2,4-disulfophenyl)-2H-tetrazolium, mono-sodium salt] (Dojindo Lab., Kumamoto, Japan), reduced to formazan dye by mitochondrial dehydrogenases. Thus, WST-8 assay was used to estimate the cytotoxicity of EGCG to VECs and VSMCs. Each cell culture with 2 × 10^4^ cells/well as an initial seeding number was treated with increasing concentrations of EGCG and then incubated with WST-8 for the last 4 hr of the culture period (24 hr) at 37 °C in the dark. In order to avoid a direct reaction between EGCG and WST-8, the culture was thoroughly washed followed by the medium exchange before adding WST-8. Parallel sets of wells containing freshly cultured, non-treated cells were regarded as the controls. Absorbance was determined at 450 nm using an ELISA reader (Spectra Max 340, Molecular Device Inc., CA, USA). The relative cell viability (% of control) was expressed as the percentage of the optical densities in EGCG-treated wells to the optical density in a non-treated well. From the cytotoxicity profile, IC_50_ value (μM) was defined as the concentration of EGCG in culture media which decreases cell viability down to 50%.

### 3.4. EGCG Treatments

EGCG (Teavigo™, ≥98% purity) was purchased from DSM Nutritional Products (Basel, Switzerland). In order to examine the effects of EGCG on the proliferation of VECs and VSMCs, both cells were seeded into well plates and then incubated in the presence of increasing concentrations (0 ~ 400 μM) of EGCG in their respective complete medium for 24 hr. Upon determining the differential effects of EGCG on the migration of VECs *vs.* VSMCs, each cell was treated with 200 μM EGCG in each complete medium for up to 36 hr.

### 3.5. Cell Proliferation Assay

Cell proliferation was found to be directly proportional to the metabolic reaction products obtained in WST-8. As describe above, the proliferation of VECs and VSMCs exposed to increasing concentrations of EGCG were determined by WST-8 assay. Both cells were treated with EGCG and then incubated with WST-8 for 4 hr followed by measuring absorbance at 450 nm.

### 3.6. Cell Migration Assay

As described in our previous studies [35,36], *in vitro* migration assays were performed. In brief, VECs or VSMCs (1 × 10^5^ cells/mL) were seeded in 4-well chambered cover-glass slide and grown to confluence overnight. Mono-layers were scraped (denuded) using a 1 mL plastic micropipette tip, and 200 μM EGCG was treated to the confluent cell layers. EGCG-treated or non-treated cells were incubated in a self-designed CO_2_ mini-incubator placed on the stage of an inverted system microscope (IX70, Olympus Optical Co., Osaka, Japan) and then visualized for cells migrating into denuded space by a CCD camera (Olympus Optical Co.) attached to the microscope. Migration of cells into denuded areas was monitored for up to 36 hr. The average area covered by migrated cells and the migration speed of an individual cell were calculated by using image processing software (MATLAB V5.3, Mathwork Inc., Natic, MA, USA).

### 3.7. Statistical Analysis

All variables were tested in three independent cultures for each experiment, and each experiment was repeated twice (n = 6). The results are reported as a mean ± standard deviation (SD) compared with the non-treated controls. A one-way analysis of variance (ANOVA), which was followed by a Tukey HSD test for the multiple comparisons, was used to detect the effects of EGCG on VECs and VSMCs. A *p* value < 0.05 was considered statistically significant.

## 4. Conclusions

In conclusion, both vascular cells were significantly affected by EGCG of which the higher concentrations (over 200 μM) inhibited the more cell proliferation. However, EGCG exerted differential migration-inhibitory activity in VECs *vs.* VSMCs. EGCG-mediated inhibition of migration was found to occur at much higher dose of EGCG in VECs as compared to VSMCs. Although this kind of peculiar response of VECs to EGCG could not be obviously explained at present, it would be suggested that EGCG might be exploited as an efficient drug for a stent due to its selective activity, completely suppressing the proliferation and migration of VSMCs, but not adversely affecting VECs migration in blood vessels.
